# Physical and Physiological Demands of Recreational Team Handball for Adult Untrained Men

**DOI:** 10.1155/2017/6204603

**Published:** 2017-03-29

**Authors:** Susana C. A. Póvoas, Carlo Castagna, Carlos Resende, Eduardo Filipe Coelho, Pedro Silva, Rute Santos, André Seabra, Juan Tamames, Mariana Lopes, Morten Bredsgaard Randers, Peter Krustrup

**Affiliations:** ^1^Research Center in Sports Sciences, Health Sciences and Human Development, CIDESD, University Institute of Maia, ISMAI, Maia, Portugal; ^2^Fitness Training and Biomechanics Laboratory, Italian Football Federation, Technical Department, Coverciano, Florence, Italy; ^3^School of Sport and Exercise Sciences, University of Rome Tor Vergata, Rome, Italy; ^4^University Institute of Maia, ISMAI, Maia, Portugal; ^5^Center of Research, Education, Innovation and Intervention in Sport, Faculty of Sport, University of Porto, Porto, Portugal; ^6^Portuguese Handball Federation, Lisbon, Portugal; ^7^Research Center in Physical Activity, Health and Leisure, Faculty of Sport, University of Porto, Porto, Portugal; ^8^Early Start Research Institute, School of Education, Faculty of Social Sciences, University of Wollongong, Wollongong, NSW, Australia; ^9^Department of Chemistry and Biochemistry, Faculty of Sciences, University of Porto, Porto, Portugal; ^10^Department of Sports Science and Clinical Biomechanics, SDU Sport and Health Sciences Cluster (SHSC), University of Southern Denmark, Odense, Denmark; ^11^Sport and Health Sciences, College of Life and Environmental Sciences, University of Exeter, Exeter, UK

## Abstract

Lack of motivation to exercise was reported as a major cause of sedentary behavior in adulthood. This descriptive study examines the acute physical and physiological demands of recreational team handball and evaluates whether it could be suggested as an exercise mode for fitness and health enhancement in 33–55-year-old untrained men. Time-motion, heart rate (HR), and blood lactate analyses were obtained from 4 recreational matches. Mean distance covered during the 60 min matches was 6012 ± 428 m. The players changed match activity 386 ± 70 times, of which high-intensity runs and unorthodox movements amounted to 59 ± 18 and 26 ± 26 per match, respectively. The most frequent highly demanding playing actions were jumps and throws. Match average and peak HR were 82 ± 6% and 93 ± 5%  HR_max_, respectively. Players exercised at intensities between 81 and 90%  HR_max_ for 47% (28 ± 14 min) and >90%  HR_max_ for 24% (14 ± 15 min) of total match time. Match average and peak blood lactate values were 3.6 ± 1.3 and 4.2 ± 1.2 mM, respectively. Recreational team handball is an intermittent high-intensity exercise mode with physical and physiological demands in the range of those found to have a positive effect on aerobic, anaerobic, and musculoskeletal fitness in adult individuals. Training studies considering recreational team handball as a health enhancing intervention are warranted.

## 1. Introduction

Exercise has been established as a cornerstone in the prevention and treatment of cardiovascular and metabolic diseases [1] and physical activity (PA) primarily prevents, or delays, several chronic conditions [2]. Yet, a third of the adults worldwide do not reach the recommended levels for daily PA [3] and only 8% of the European population report exercising or playing sport regularly, whereas 20% indicate lack of interest or motivation as one of the causes for not practicing sport more frequently [4]. Physical inactivity was shown to be responsible for 5.3 million deaths per year worldwide [5]; hence, it is of paramount importance to identify more types of exercise that can meet the PA guidelines, while keeping the participants motivated and, preferably assuring long-term adherence.

Most studies addressing the effect of physical training on health have focused on the effects of continuous aerobic exercise training on cardiovascular and metabolic fitness or on the effects of strength training on the risk of falls and fractures related to changes in muscle function and bone strength. Fewer studies have investigated the health effects of regular participation in a variety of team sports, which involve aerobic high-intensity training along with anaerobic training, including sprints and specific actions with a high impact on the musculoskeletal system [6]. Most of these studies have focused on recreational soccer played as small-sided games, showing a high aerobic component with mean heart rates (HR) of 80–85% of maximum HR (HR_max_). Additionally, the activity profile includes multiple high-intensity runs, sprints, turns, jumps, and tackles, which provide high demands on the anaerobic metabolism and high impact on muscles and bones [7–10].

Short-term recreational soccer training programmes resulted in marked improvements in cardiovascular, metabolic, and musculoskeletal health markers, with low perceived exertion (3.9 of 10). These positive broad-spectrum effects are reported for different populations, inducing a marked reduction in the risk of developing lifestyle chronic diseases [6].

Competitive team handball, as most of the field and court team sports (i.e., soccer, rugby, basketball, and futsal, resp.) [11–14], is a high-demanding intermittent exercise that primarily uses the aerobic metabolism, interspersed by high-intensity actions that greatly tax the anaerobic pathway due to numerous high-intensity displacements and actions that occur throughout the game [15–17]. The relative workload during the matches is 65–80% of VO_2_max, the total distance covered per match ranges from 2600 to 4700 m, consisting mostly of walking and jogging (~70% of match time), HR_mean_ is 80%  HR_max_, a high number of activities changes (600–1500 per match), and a postmatch blood lactate of 3–11 mM was reported. The activity profile differs across playing positions [15, 17–20] and competitive level [18] and neuromuscular fatigue occurs throughout the game [19, 21, 22]. The team handball activity profile is comparable to what has been reported in small-sided versions of soccer, basketball, and futsal [12, 23, 24] with similar total distance covered but higher demands imposed on the upper body [25]. In competitive team handball, there are many high-intensity actions like jumps, shots, and tackles related to one-on-one situations. Such actions result in high bone and muscle impact along with a higher injury risk. However, it has not yet been investigated to what extent these actions occur during recreational team handball. Thus, it is of interest for health issue to ascertain to what extent recreational team handball practice has enough cardiovascular and musculoskeletal impact to potentially induce health and fitness adaptations, similar to recreational soccer. Therefore, the purpose of this study is to describe the physical and physiological demands of recreational team handball in untrained men. We hypothesized that recreational team handball is a high-intensity exercise mode, characterized by frequent jumps, throws, stops, changes of directions, and one-on-one situations functional in eliciting physical and physiological demands in the domain reported to enhance aerobic, anaerobic, and musculoskeletal fitness.

## 2. Materials and Methods

### 2.1. Subjects

Fifteen participants (13 outfield players and 2 goalkeepers; 42.0 ± 7.1 years; height 179.4 ± 7.3 cm; body mass 98.3 ± 9.6 kg; fat mass 22.0 ± 5.2%; VO_2_ peak 40.2 ± 7.0 mL·min·kg^−1^; Yo-Yo intermittent endurance level 2 test (YYIE2) 351 ± 149 m; systolic blood pressure 131 ± 11 mmHg; diastolic blood pressure 75 ± 10 mmHg; 62 ± 8 b·min^−1^  HR_resting_; 177 ± 10 b·min^−1^  HR_max_) accepted to participate in this study. They had played team handball for 19 ± 3 (12–22) years and their training history was an average of 2–7 training sessions, performing a total of 8 ± 3 (2–14) hours per week but had no record of regular PA for the last 13 ± 7 (4–26) years.

### 2.2. Experimental Design

The activity profile and the physiological loading (HR, blood lactate, and rating of perceived exertion [RPE]) were analyzed during 4 training sessions, consisting of 60 min (7v7) recreational team handball matches, in fifteen untrained men with previous experience with this sport. The matches were interspersed by a 10-min half-break and were preceded by a standardized 15-min warm-up consisting of 5 min of self-paced jogging, running at progressively increasing speeds, changes of direction, arm rotation with the ball, and technical ball drills (passes, throws to goalie with a goalkeeper, and one-on-one situations). No time-out periods were allowed unless for players' physical assistance (e.g., injuries). The average number of players per match was 14, meaning that substitutions were seldom needed. Analyses were only performed on players who played the entire match duration. Participants were previously familiarized with all testing procedures before the commencement of this study during specific sessions. Additionally, they were informed of the aims and the experimental risks of the study, before giving their informed written consent to participate. This study was conducted in accordance with the Declaration of Helsinki and ethical approval was provided by the local Institutional Review Board.

### 2.3. Experimental Procedures

Thirty video-film recordings (22 outfield players and 8 goalkeepers) were analyzed to establish match activity profile according to the methods previously described by Póvoas et al. [26]. Outfield players' displacements were coded into eight locomotor categories defined according to Randers et al. [27] and considering handball's specific movements. Locomotor categories and the mean velocity of each category were determined by detailed analysis of match images using court-lines as reference during video replay analysis, as follows: (1) standing still (0 km·h^−1^), (2) walking (5 km·h^−1^), (3) jogging (10 km·h^−1^), (4) fast running (15 km·h^−1^), (5) sprinting (21 km·h^−1^), (6) backwards movement (5 km·h^−1^), (7) sideways medium-intensity movement (5 km·h^−1^), and (8) sideways high-intensity movement (14 km·h^−1^). The distance covered in each category equaled the product of the total time and the mean speed for that activity. High-intensity activities equaled the sum of categories 4, 5, and 8, low-intensity activities were the sum of categories 1, 2, 3, 6, and 7, and unorthodox movements were the sum of categories 6, 7, and 8. In addition, five types of specific handball playing actions were also studied for the outfield players: (1) jumps, (2) throws, (3) stops when preceded by high-intensity activities, (4) changes of direction, and (5) one-on-one situations. The playing actions analyzed for the goalkeepers were (1) jumps, (2) stops, (3) changes of direction, and (4) defenses. For each locomotor category, the following was determined: percentage of total time and distance, duration, distance, and frequency. All analyses were performed by one experienced observer and the first and the second halves of each match were analyzed in a random order [20, 26].

During the matches the players wore a GT3X+ 30 Hz accelerometer (Actigraph, Pensacola, Florida, USA) to track PA pattern and exercise intensity. The device was used on the hip near the center of gravity, underneath clothing, and secured with an elastic belt. Data download, cleaning, and analyses were performed using the manufacturer software (Actilife, version 6.6.3 for windows). The average PA expressed as mean counts per minute (cpm, corresponding to the counts obtained divided by the number of minutes of wear time per match) was calculated. PA intensities were expressed in minutes and were calculated based on recommended vector magnitude cut points [28]. Time spent in moderate-to-vigorous physical activity (MVPA) was determined to access the matches' contribution for players meeting the PA guidelines (adults aged 18–64 years ≥ 150 minutes·week^−1^ of MVPA intensity). Time in vigorous PA was also calculated to access the proportion of achievement of the guidelines of ≥75 minutes·week^−1^ of vigorous PA [29].

HR recordings (167) of fifteen players (30 wings, 70 backcourt players, 11 pivots, and 56 goalkeepers) were registered, beat-by-beat, using HR monitors (Firstbeat Technologies Ltd., version 4.5.0.2, Jyväskylä, Finland) during fourteen matches. Considered HR zones were ≤50, 51–60, 61–70, 71–80, 81–90, and 91–100%  HR_max_.

To determine blood lactate concentrations [Blac], capillary blood samples (30 *μ*L) of thirteen players (*n* = 28 records; 4 matches) were drawn from the right earlobe, at rest and at 5, 10, 15, 20, and 25 min and at the end of the first and of the second halves. A portable electroenzymatic lactate device analyser (Lactate ProTM, Quesnel, Canada) was used.

RPE was recorded 30-min after the end of all analyzed matches, in order to ensure that the rating reflected the whole match and not only the last period, using Borg's RPE category ratio 10-scale (CR10) modified by Foster et al. [30].

Sweat loss during a match was determined according to Póvoas et al. [26]. The matches were held under neutral temperature (17–22°C) and humidity conditions (50–70%) (Extech Digital Hygrometer 445715; Grainger, New York, NY, USA).

Body mass and percentage of body fat were determined by bioimpedance analysis (Tanita Inner Scan digital, BC532, Amsterdam, The Netherlands). Participants performed an incremental treadmill test [31] until voluntary exhaustion to determine peak oxygen consumption (Quasar-Med, Nussdorf, Germany). Expired respiratory gas fractions were measured using an open circuit breath-by-breath automated gas-analysis system (Cortex, Metalyzer, 3B, Leipzig, Germany).

HR_max_ was considered as the highest HR value resulting from the incremental treadmill test or the YYIE2 [32] using short-range telemetry (Firstbeat Technologies Ltd., version 4.5.0.2, Jyväskylä, Finland). The YYIE2 consists of 2 × 20 m shuttle runs interspersed by 5 s active recovery periods, with increasing running speeds as the test progresses [32].

Players and goalkeepers were analyzed separately due to pronounced differences in the physical and physiological profile [20].

All testing was performed at the end of the afternoon, and the participants were advised to eat a normal diet, including carbohydrates, the day before testing and to eat lunch at least 2 hours before testing and to be properly hydrated.

### 2.4. Statistical Analyses

Results are presented as means ± standard deviation (SD) and range. Reliability of all variables was estimated according to Póvoas et al. [26] using a test-retest procedure after seven days in a random subsample of two matches for seven of the participants. The intraclass correlation coefficient (ICC_3,1_) used to calculate relative reliability of all variables [33, 34] was considered as excellent (*R* > 0.80) [35].

Differences between [Blac], time-motion and HR variables during the two halves were assessed by Student's paired *t*-test with 95% Confidence Interval (95% CI). Differences between high and low-intensity activities were determined by Student's unpaired *t*-test with 95% CI. Practical significance was assessed by calculating Cohen's* d* effect size and interpreted as suggested by Batterham and Hopkins [36] (*d*, ≤0.2: trivial, >0.2–0.6: small, >0.6–1.2: moderate, >1.2–2.0: large, and >2.0–4.0: very large). The differences between [Blac] at baseline and in each half of the matches were examined by repeated measures analysis of variance (ANOVA). The Bonferroni test was used for multiple comparisons with 95% CI. Effect size was calculated using partial eta-squared (*η*_*p*_^2^) and interpreted as small (≥0.01), medium (≥0.06), or large (≥0.14) [37]. Statistical Package for the Social Sciences (SPSS Inc., version 22.0) was used for all analyses. Statistical significance was set at *P* ≤ 0.05.

## 3. Results

Match activity data are presented in Table 1.

Total distance covered during the average 60 ± 3 min handball matches was 6012 ± 428 m. The players changed match activity 386 ± 70 times, and the sum of high-intensity runs, and unorthodox movements were 59 ± 18 and 26 ± 26 per match, respectively. The most frequent highly demanding playing actions were jumps and throws (Table 2).

**Figure 1:**
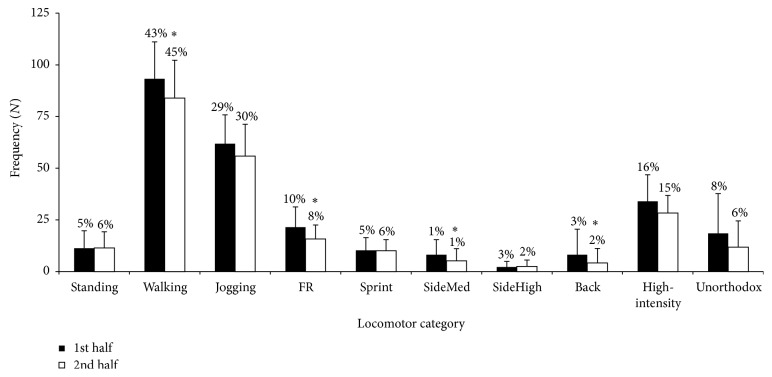
Absolute and relative number of occurrences in each locomotor category in the first and second halves of recreational team handball matches (means ± SD). FR: fast running; Back: backwards movement; SideMed: sideways medium-intensity movement; SideHigh: sideways high-intensity movement; high-intensity (sum of fast running, sprinting, and sideways high-intensity movement); unorthodox (sum of backwards, sideways medium-intensity, and sideways high-intensity movement); ^*∗*^*P* ≤ 0.01 significantly different from the first half of the match.

Absolute and relative number of occurrences for each locomotor and intensity category in each of the halves of the match are presented in Figure 1.

Total number of occurrences (217 ± 47 versus 189 ± 45; 95% CI 11,44; *P* = 0.02; *d* = 0.83) as well as the number of occurrences in walking (95% CI 2,17; *d* = 0.59), fast running (95% CI 3,9; *d* = 0.94), sideways medium-intensity (95% CI 1,5; *d* = 0.68), and backwards running (95% CI 1,7; *d* = 1.53) decreased in the second half (*P* ≤ 0.01; Figure 1). Moreover, the number of stops (95% CI 0,3; *d* = 0.44) and changes of direction (95% CI 0,3; *d* = 0.60) decreased from the first to the second halves (*P* ≤ 0.05; Table 2), as well as the total distance covered (3133 ± 233 versus 2944 ± 251 m, first versus second halves; 95% CI 96,282; *P* < 0.01; *d* = 0.95). A significant decrement in distance covered in fast running (377 ± 273 versus 287 ± 240 m, 95% CI 44,135; *P* < 0.01; *d* = 0.98) and in low-intensity activities (558 ± 301 versus 458 ± 254 m, 95% CI 23,177; *P* < 0.01; *d* = 0.63) was shown during the second half.

Outfield player's PA intensities measured by accelerometer during the matches are presented in Table 3. PA guidelines per match in MVPA and vigorous intensity were 27 ± 7 (4–39)% and 10 ± 9 (0–36)%, respectively.

Peak HR during the matches was 167 ± 13 b·min^−1^ and the mean HR was 148 ± 14 b·min^−1^ (93 ± 5 and 82 ± 6%  HR_max_, resp.) for outfield players.

Outfield players exercised at intensities between 81 and 90%  HR_max_ for 28 ± 14 min (47% of total match time) and above 90%  HR_max_ for 14 ± 15 min (24%). Only 11% of total match time (9 min) was performed at HRs equal or lower than 70%  HR_max_.

**Figure 2:**
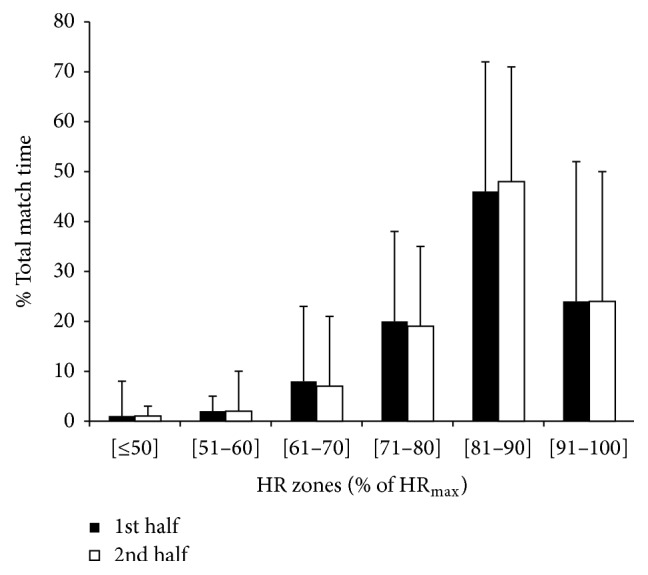
Percentage of effective match time spent at different interval percentages of players' maximal heart rate (HR_max_) in the first and second halves of the recreational team handball matches (means ± SD).

The percentage of match time spent by players in the here considered HR zones in both halves is presented in Figure 2.

**Figure 3:**
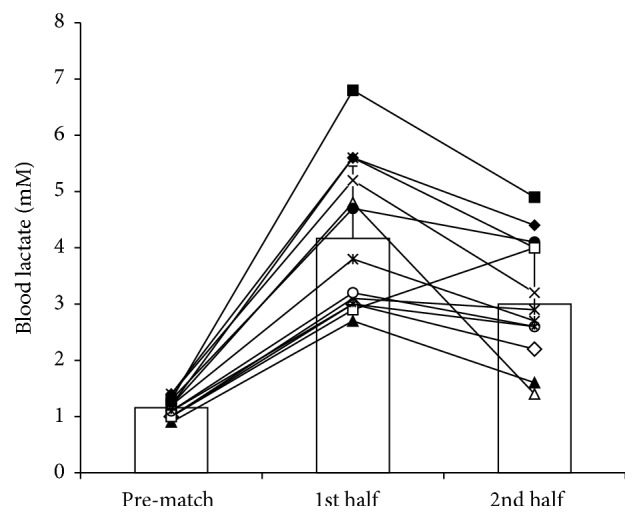
Average (SD) and individual blood lactate values before (baseline) and during the first and second halves of the recreational team handball matches.

Average and peak [Blac] were 3.6 ± 1.3 (1.4–6.8) mM and 4.2 ± 1.2 mM (2.7–6.8), respectively. Values increased significantly (*P* ≤ 0.01) from baseline (1.2 ± 0.2; 0.9–1.4 mM; 95% CI −3.9, −2.2) to the first (4.2 ± 1.3; 2.7–6.8 mM; 95% CI −2.6, −1.0) and second (3.0 ± 1.1; 1.4–4.9 mM) halves (*η*_*p*_^2^ 0.793; *P* = 0.01) (Figure 3). A significant decrease in [Blac] was shown from the first to the second half (95% CI −0.4, −2.0; *P* = 0.01).

Postmatch body mass loss was 1.1 ± 0.5 (0.6–2.4) kg corresponding to 1.2 ± 0.4% (0.7–2.4) of body mass. Match fluid intake was 0.1 ± 0.3 (0.0–0.9) L with a total fluid loss of 1.3 ± 0.5 (0.6–2.4) L corresponding to 1.3 ± 0.5 (0.7–2.4)% of the body mass. Outfield players' postmatch RPE was 7.6 ± 1.3 (2–10) AU (very hard).

Goalkeepers performed an average of 35 ± 8 jumps, 16 ± 6 stops, 26 ± 6 changes of direction, and 20 ± 4 defense actions. Peak and mean HRs were 85 ± 11 and 72 ± 11%  HR_max_, respectively. Goalkeepers exercised at intensities between 81 and 90%  HR_max_ for 22 ± 23% of the total match time and only 6 ± 12% above 90%  HR_max_. They played at intensities equal or below 70%  HR_max_ for 44% of total match time.

Goalkeepers' time performed at light, moderate, and vigorous activity was 30.32 ± 10.44 (9.00–43.83) min, 21.67 ± 5.75 (10.17–29.33) min, and 2.98 ± 3.42 (0.00–10.00) min, respectively.

Sedentary time was 2.82 ± 1.70 (0.17–6.33) min and zeros count was 5 ± 10 (0–34) min.

Match contribution for MVPA and vigorous intensity PA guidelines was 16 ± 5 (8–26)% and 4 ± 5 (0–13)%, respectively.

Average [Blac] was 1.1 ± 0.1 (0.9–1.1) mM and postmatch RPE was 4.8 ± 2.5 (1–8) AU (hard).

The goalkeepers' demands were significantly different (*P* < 0.05) from the outfield players.

## 4. Discussion

This is the first paper examining the physical and physiological demands of recreational team handball in untrained adults (33–55 years) with previous experience with this sport. No study had previously described the potential of this sport in its recreational mode for inducing positive cardiovascular and musculoskeletal adaptations and, thus, health and physical fitness enhancement. The results showed that recreational team handball is an intense intermittent activity with high aerobic and anaerobic demands. Indeed, outfield players were involved in frequent highly demanding specific actions such as jumps, throws, stops, changes of direction, one-on-one situations, and unorthodox movements. These match actions promoted cardiovascular strain in the range of that reported to positively affect aerobic fitness in healthy adult individuals [38]. As in previous studies [20], goalkeepers' demands were significantly lower (*P* < 0.05) from the outfield players. These results confirmed the original work hypothesis suggesting recreational team handball as a valid fitness and health enhancing exercise mode.

Team sports played at competitive level are ideally supposed to induce higher physiological and neuromuscular demands than when played at recreational level. The actual difference is the cause of players' training status, body make-up, and genetic and associated modification in playing rules. In this study, recreational outfield handball players spent a lower percentage of total match time standing still than elite team handball players (4 versus 43%) but spent a higher percentage of match time walking (71 versus 35%). Interestingly, in this study players covered more total distance (6012 versus 4370 m) and performed less activity changes (388 versus 825) than elite team handball players [26]. These results are probably due to the much smaller number of playing time stoppages (0.4 versus 13.7, recreational versus elite) and substitutions performed during the recreational matches, since in elite team handball matches several situations can interrupt the match time [26]. Percentage of total match time spent in high-intensity activities (6%) was similar to that reported in elite team handball matches (4%) and in other team sports such as soccer [39], basketball [11, 40], and futsal [12]. However, a higher number of high-demanding movements, actions, and unorthodox movements were performed by recreational team handball players. It has been suggested that unorthodox modes of motion such as backwards and sideways running accentuate the metabolic load eliciting elevated levels of energy expenditure [41, 42]. This may partly explain the high cardiovascular strain imposed on the team handball players.

Despite recreational players' higher number of throws and jumps, the number of stops, changes of direction, and one-on-one situations were lower than those reported in elite team handball players. This difference is probably due to a recreational players' preventive action towards injuries. In team handball, the number of injuries related to body contact or side-cutting manoeuvres is quite high [43–45]. Considering that the participants were former experienced competitive team handball players, it is expectable that they would avoid match situations in which the probability of injury was high. Interestingly, jumps and throws accounted for 60% of the total 54 playing actions registered, unlike elite team handball matches in which the same percentage was due to stops and changes of direction [26]. Thus, the frequent jumps, throws, changes of directions, one-on-one situations, and unorthodox movements may constitute a viable way to stress musculoskeletal adaptations in recreational players. Additionally, the spontaneous reduction of match collision further promotes the interest of recreational team handball for health issues as injury rate is likely reduced compared to the competitive version. Noteworthy is also the total number of high-demanding actions being half the number performed by elite handball players (54 versus 104). Nevertheless, it should be taken into account that the recreational matches lasted an average of 60 ± 3 min, while the elite matches lasted 73 ± 5 min. The recreational team handball matches suggest their interest for the development of the neuromuscular performance of lower and upper limbs differently from small-sided soccer and futsal played at recreational level. Interestingly, the reported difference in activity profile and diversity in external load further support the validity of addressing recreational team sports and of this study research design.

In the analyzed matches, mean HR value was 82%  HR_max_, which is similar to the intensity found in elite team handball players (82%  HR_max_) [26], recreational soccer players (80–85%  HR_max_) [6], and futsal players (83.5 ± 5.4%  HR_max_) [46]. This HR intensity is markedly higher than the minimum suggested for cardiovascular fitness by the American College of Sports Medicine (55–65%  HR_max_) [29]. Based on the results from recreational team sports and soccer studies, in particular [6], altogether, the physical and physiological loading imposed by team handball matches may induce positive metabolic, cardiovascular, and musculoskeletal changes in healthy individuals. Thus, training studies using recreational team handball as an exercise mode are warranted. Indeed, this sport has been reported to be played by ~19 million worldwide [17], so the potential for generalization is appealing not only in former team handball trained players but also for fans. Thus, future studies should consider the importance of training expertise.

Besides the impact on the legs muscles, which also occurs as in other team sports such as soccer, recreational team handball matches additionally greatly involve the core and upper body [25]. Given this, a greater number of muscles and bones are involved during recreational team handball, which may result in a broader range of health benefits, since adaptations are specific to the stimulated structure [47].

Although accelerometer data is considered the gold-standard tool to estimate exercise intensity in health-promoting interventions, there is strong evidence that high HR (i.e., HR above 80%  HR_max_) has a positive impact on cardiovascular health changes preventing cardiovascular diseases [29]. Furthermore, HR values of the outfield players were above 80%  HR_max_ for 42 min and above 90%  HR_max_ for 14 min (71% and 24% of total match time, resp.) which is considered sufficient to cause marked improvements in cardiorespiratory fitness, systolic blood pressure, and glucose tolerance, increasing the overall health profile [7, 48]. Moreover, even though the players were standing still or walking during a relevant part of the match time (75%), the cardiovascular strain was high. Nonetheless, surprisingly, accelerometer data showed only 27% of total match time in MVPA intensity and 10% in vigorous intensity, per match session. These results show somewhat a pitfall of accelerometer data and of reported cut-off points in characterizing the overall physical demands of this type of activity. A comprehensive description of team handball demands can only be achieved with the complementary use of time-motion and HR analysis. Indeed, several high-intensity activities such as accelerations, decelerations, stops, throws, jumps, changes of directions, and one-on-one situations may induce high prolonged HR, without significantly impacting the time spent or distance covered in the locomotor category activities performed [26].

Caution should be taken concerning the possible health effects of recreational handball in goalkeepers, since HRs were above 80%  HR_max_ for 17 min (i.e., 28% of total match time) with only 4 min (6%) being spent above 90%  HR_max_. Nevertheless, match activity profile showed that players in this playing position performed 97 high-demanding actions such as jumps, stops, changes of direction, and defenses per match that can positively impact on the musculoskeletal system [6]. For the outfield players, the percentage of total match time spent at intensities above 80%  HR_max_ was higher than for elite team handball players (71 versus 53%) [26]. As mentioned before, this is probably due to the very small number of playing time stoppages and substitutions performed during the matches. Blood lactate values were similar to elite team handball players but lower than elite [39, 49] and recreational soccer players [10].

Despite the overall high intensity of the matches and the decrease shown in the second half in the frequency and distance covered in some of the locomotor categories (small to large effect), game actions (small effect), and [Blac] values (large effect), both the outfield players and the goalkeepers seem to be able to sustain similar cardiovascular load during the entire match (60 min). In fact, there were no significant differences in mean HR or in percentage of total match time spent above 80%  HR_max_ between the halves.

Unlike recreational soccer small-sided games and recreational futsal [46], in which participants reported 3.9 ± 1.8 and 4.1 ± 0.8 RPE [50], respectively, recreational team handball players felt they exerted themselves “very hard” (7.6 ± 1.3) during the matches. This result is in accordance with the activity profile and the physiological demands of the analyzed matches and indicates that former experienced competitive players probably more accurately evaluate the intensity of the activity and are able to reach near maximal efforts [46]. Additionally, it can be speculated that the musculoskeletal strain related to the frequent body contact imposed by team handball matches may result in higher RPE from the players. Interestingly, during basketball drills, increments in RPE were reported when reducing the player number in the court, being the 5v5 (actual basketball) the less demanding condition compared to 3v3 and 2v2 (i.e., 4.5 ± 1.8, 5.8 ± 1.1, and 6.8 ± 1.5, resp.). However, despite the use of upper limbs during basketball (i.e., 5v5) the RPE was lower during the simulated games [24]. Differences in RPE may be due to playing rules, aerobic fitness, and skills of the recreational players [46].

Given that the participants in this study had prior experience with team handball, future studies should investigate whether the demands are equally high for participants with no or little prior experience in this team sport.

In conclusion, this study results show that recreational team handball is an intermittent high-intensity exercise mode, potentially useful as a training intervention for the development of cardiovascular and musculoskeletal fitness in male adult former handball players. The reported data are in line with previously published research that addressed recreational soccer and futsal widening the range of health and fitness beneficial exercise modes for the general population. Thus, considering that available exercise options are not interesting enough for all and, hence, effectively decreasing physical inactivity-induced morbidity and mortality [2] and that recreational team handball playing could be an appealing exercise mode for a relevant number of former players and fans worldwide [17], training studies considering this sport as a health enhancing intervention are warranted.

## Figures and Tables

**Table 1:** Activity profile of recreational team handball players during the match (means ± SD). Freq: frequency; FR: fast running; Back: backwards movement; SideMed: sideways medium-intensity movement; SideHigh: sideways high-intensity movement; intensity categories: high (sum of fast running, sprinting, and sideways high-intensity movement); low (sum of standing still, walking, jogging, backwards, and sideways medium-intensity movement); unorthodox movements (sum of backwards, sideways medium-intensity, and sideways high-intensity movement); ^*∗*^*P* < 0.01 significantly different from high-intensity movements.

	Locomotor categories								Intensity categories		Unorthodox movements
	Standing	Walking	Jogging	FR	Sprint	SideMed	SideHigh	Back	High	Low	
Freq (*N*)	22 ± 14	171 ± 29	114 ± 25	35 ± 15	19 ± 11	11 ± 8	4 ± 5	10 ± 18	59 ± 18	329 ± 65	26 ± 26
Freq (%)	5 ± 3	44 ± 2	30 ± 6	9 ± 4	5 ± 3	3 ± 2	1 ± 1	2 ± 3	15 ± 5	85 ± 5	6 ± 5
Mean duration (s)	7.4 ± 3.9	15.5 ± 3.4	5.2 ± 1.0	3.7 ± 1.5	2.7 ± 0.6	1.3 ± 0.4	1.6 ± 1.1	3.3 ± 1.4			
Mean distance (m)		20.2 ± 4.5	13.7 ± 2.6	15.2 ± 6.4	15.9 ± 3.3	1.6 ± 0.5	6.1 ± 4.1	4.6 ± 1.9			
Fraction of total time (%)	4 ± 2	71 ± 6	16 ± 5	4 ± 3	1 ± 1	1 ± 1	0 ± 0	2 ± 4	6 ± 3	94 ± 3^*∗*^	3 ± 4
Fraction of total distance (%)		57 ± 6	26 ± 7	10 ± 6	5 ± 2	0 ± 1	0 ± 0	1 ± 3	15 ± 6	85 ± 6	2 ± 3
Total time (s)	153 ± 90	2578 ± 209	593 ± 178	155 ± 120	54 ± 26	22 ± 24	7 ± 11	68 ± 128	216 ± 125	3414 ± 136^*∗*^	97 ± 150
Total distance covered (m)		3400 ± 233	1600 ± 466	585 ± 425	305 ± 150	34 ± 45	29 ± 43	69 ± 143	918 ± 438	5094 ± 372	122 ± 172

**Table 2:** Game actions and time-outs frequency during recreational team handball matches. Values are presented for the total match and for each half (means ± SD). ^*∗*^*P* ≤ 0.05 significantly different from the first half of the match.

Game actions	First half	Second half	Total
Jumps	8.1 ± 5.0	9.2 ± 5.9	17.3 ± 10.3
Throws	7.0 ± 3.9	7.8 ± 4.7	14.8 ± 7.7
Stops	3.3 ± 3.1	2.1 ± 1.7^*∗*^	5.4 ± 4.0
Changes of direction	4.5 ± 3.7	2.9 ± 2.6^*∗*^	7.4 ± 5.7
One-on-one situations	4.7 ± 2.8	4.7 ± 2.8	9.4 ± 5.5

Time-outs	0.2 ± 0.7	0.2 ± 0.4	0.4 ± 0.8

**Table 3:** Player's physical activity intensities measured by accelerometer [means ± SD (range)] during recreational team handball matches. Cmp: counts per minute (min).

	Mean ± SD (min–max)
Zeros (min)	5.52 ± 9.78 (0.00–39.67)
Sedentary (min)	1.98 ± 1.87 (0.17–10.33)
Light (min)	19.42 ± 6.68 (6.50–35.00)
Moderate (min)	33.33 ± 9.45 (5.33–53.33)
Vigorous (min)	7.26 ± 6.75 (0.00–26.83)
Vector magnitude (cpm)	4367 ± 970 (1674–6518)
Step counts (*N*)	6056 ± 1521 (1217–8613)
Steps per minute (*N*)	91 ± 16 (33–116)
